# Supplementary material for: Airway Prevotella promote TLR2-dependent neutrophil activation and rapid clearance of Streptococcus pneumoniae from the lung
Source: Nat Commun. 2022 Jun 9;13:3321. doi: 10.1038/s41467-022-31074-0 (PMC9184549; doi:10.1038/s41467-022-31074-0)
Supplement: Supplementary file 1 — Supplementary Information [file 41467_2022_31074_MOESM1_ESM.pdf]

**Airway *Prevotella* promote TLR2-dependent neutrophil activation and rapid clearance of *Streptococcus pneumoniae* from the lung**

## SUPPLEMENTARY INFORMATION

## Supplementary Figures


**Supplementary Figure 1**

**Supplementary Figure 2**

**Supplementary Figure 3**

**Supplementary Figure 4**

**Supplementary Figure 5**

**Supplementary Figure 6**

**Supplementary Figure 7**

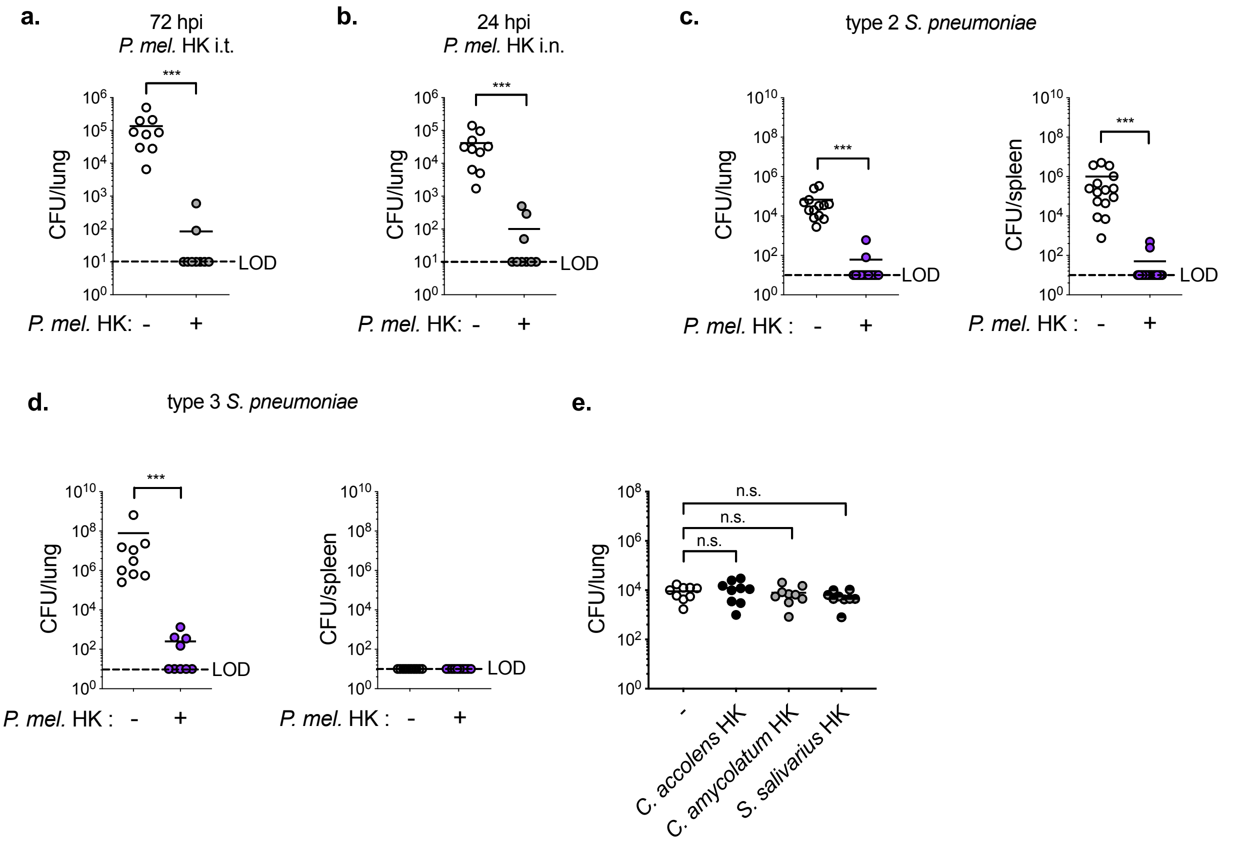


**Supplementary Figure 1.** Exposure to airway *P. melaninogenica* (*P. mel.*) heat-killed (HK) i.t. or i.n. induces clearance of *S. pneumoniae***. a** Lung type 2 *S. pneumoniae* burdens in mice exposed to PBS (-) or *P. melaninogenica* (*P. mel.*) strain 25845 HK i.t. prior to 72 h *S. pneumoniae* infection, 5x10$^{6}$ CFU/mouse (n= 9 mice/group). **b** Lung *S. pneumoniae* burdens in mice exposed to PBS (-) or *P. mel.* HK i.n. prior to 24 h *S. pneumoniae* infection (n= 10 mice/group). **c - d** Tissue burdens of type 2 *S. pneumoniae* strain D39 (n= 13 mice/group) (**c**) or type 3 *S. pneumoniae* strain ATCC 6303 (n= 9 mice/group) (**d**) following exposure to PBS (-) or *P. mel.* HK i.t. prior to 24 h *S. pneumoniae* infection, 5x10$^{6}$ CFU/mouse. **e** Lung type 2 *S. pneumoniae* burdens in mice exposed to PBS (-), *C. accolens* HK, *C. amycolatum* HK, or *S. salivarius* HK i.t. (10$^{7}$ CFU equivalents/mouse each) i.t. prior to 24 h *S. pneumoniae* infection, 5x10$^{6}$ CFU/mouse (n= 9 mice/group). LOD = limit of detection. Data are pooled from 3

independent experiments, displayed as mean ± SEM. For (**a**), ***$p<.0001$, two-tailed Mann-Whitney U test, (**b**) ***$p<.0001$, two-tailed Mann-Whitney U test, (**c**) ***$p<.0001$, two-tailed Mann-Whitney U test, (**d**) ***$p<.0001$, two-tailed Mann-Whitney U test, (**e**) from left to right $p>.9999$, $p>.9999$, $p=.5156$, Kruskal-Wallis with Dunn's *post-hoc* test. Source data are provided as a Source Data file.

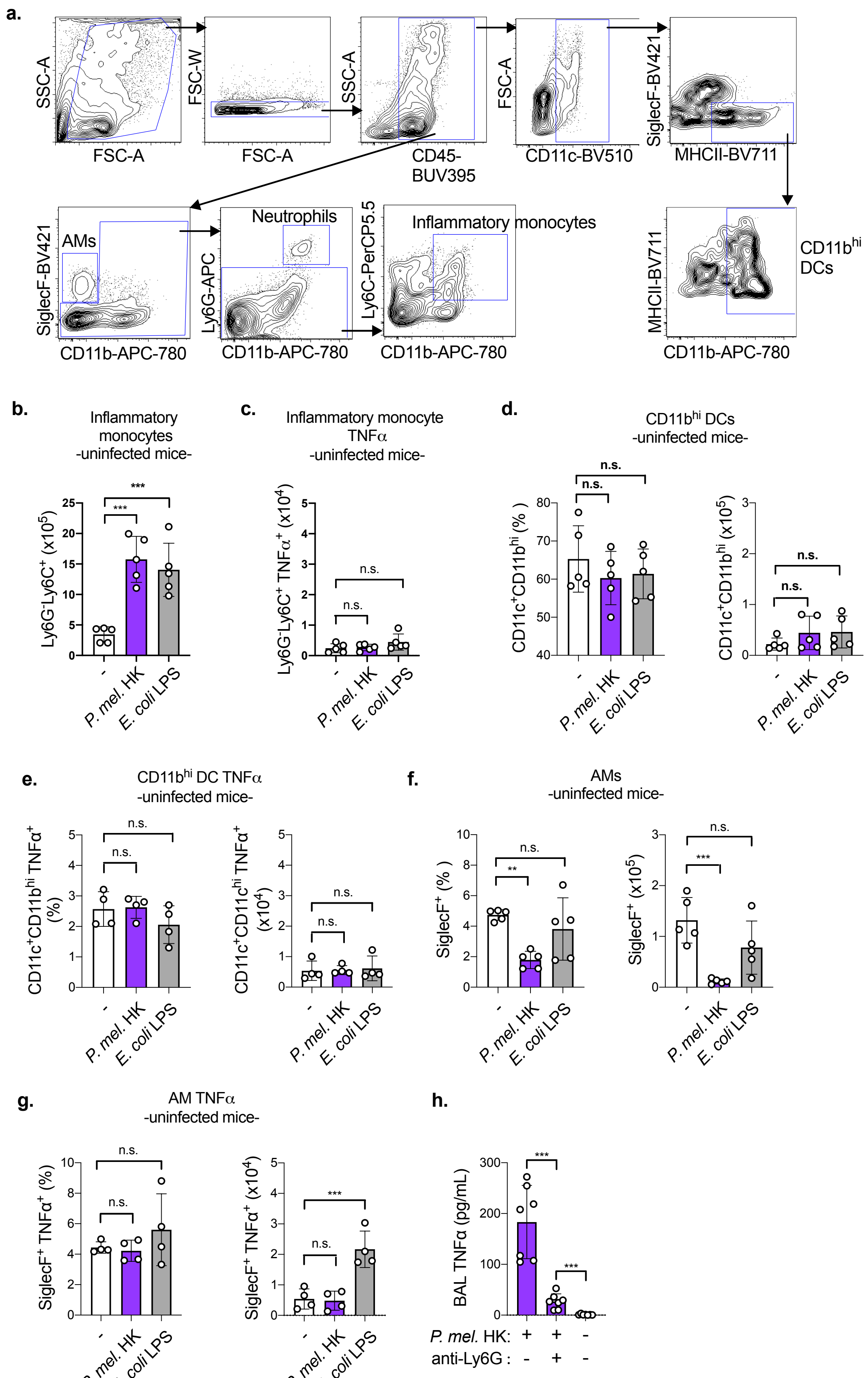
a.
SSC-A
FSC-A
FSC-W
FSC-A
SSC-A
CD45-BUV395
FSC-A
CD11c-BV510
SiglecF-BV421
MHCII-BV711
SiglecF-BV421
AMs
CD11b-APC-780
Neutrophils
Ly6G-APC
CD11b-APC-780
Inflammatory monocytes
Ly6C-PerCP5.5
CD11b-APC-780
MHCII-BV711
CD11b-APC-780
CD11b^hi DCs
b.
Inflammatory monocytes -uninfected mice-
Ly6G⁻Ly6C⁺ (x10⁵)
***
***
-
P. mel. HK
E. coli LPS
c.
Inflammatory monocyte TNFα -uninfected mice-
Ly6G⁻Ly6C⁺ TNFα⁺ (x10⁴)
n.s.
n.s.
d.
CD11b^hi DCs -uninfected mice-
CD11c⁺CD11b^hi (%)
CD11c⁺CD11b^hi (x10⁵)
n.s.
e.
CD11b^hi DC TNFα -uninfected mice-
CD11c⁺CD11b^hi TNFα⁺ (%)
CD11c⁺CD11c^hi TNFα⁺ (x10⁴)
n.s.
f.
AMs -uninfected mice-
SiglecF⁺ (%)
SiglecF⁺ (x10⁵)
**
***
n.s.
g.
AM TNFα -uninfected mice-
SiglecF⁺ TNFα⁺ (%)
SiglecF⁺ TNFα⁺ (x10⁴)
n.s.
***
h.
BAL TNFα (pg/mL)
***
***
P. mel. HK: + + -
anti-Ly6G : - + -

**Supplementary Figure 2.** Lung myeloid cell responses to *P. melaninogenica* (*P. mel.*) lung exposure**. a** Flow cytometry gating strategy defining neutrophils, alveolar macrophages (AMs), inflammatory monocytes, and CD11b$^{hi}$ dendritic cells (DCs) from single cell lung populations. This gating strategy was used for all flow cytometry analysis. **b - g** Total cell number of inflammatory monocytes (**b**), inflammatory monocyte TNF$\alpha$ (**c**), percentage and total cell number of CD11b$^{hi}$ DCs (**d**), CD11b$^{hi}$ DC TNF$\alpha$ (**e**), AMs (**f**), and AM TNF$\alpha$ (**g**) detected by intracellular flow cytometry in mice treated with PBS (-), *P. mel.* strain 25845 heat-killed (HK), or *E. coli* lipopolysaccharide (LPS) i.t. for 24 h. **h** Bronchoalveolar lavage (BAL) TNF$\alpha$ detected in mice with or without exposure to *P. mel.* HK i.t. or anti-Ly6G antibodies (200 $\mu$g/mouse) i.p. for 24 h. Data are representative from one of four independent experiments with n= 5 mice/group (**b - g**) or pooled from two independent experiments with n= 3 mice/group (replicate 1) or n= 4 mice/group (replicate 2) (**h**), displayed as mean ± SEM. For (**b**) from left to right ****p*=.0002, ****p*=.0007, one-way ANOVA with Dunnett's *post-hoc* test, (**c**) from left to right *p*=.9681, *p*=.1850, one-way ANOVA with Dunnett's *post-hoc* test, (**d**) from left to right *p*=.4835, *p*=.6292, *p*=.3598, *p*=.3099, one-way ANOVA with Dunnett's *post-hoc* test, (**e**) from left to right *p*=.9842, *p*=.3316, *p*=.3396, *p*>.9999, one-way ANOVA with Dunnett's *post-hoc* test, (**f**) from left to right ***p*=.0052, *p*=.4202, ****p*=.0009, *p*=.0968, one-way ANOVA with Dunnett's *post-hoc* test, (**g**) from left to right *p*=.9676, *p*=.4429, *p*=.0968, ****p*=.0009, one-way ANOVA with Dunnett's *post-hoc* test, (**h**) ****p*<.0001, one-way ANOVA with Dunnett's *post-hoc* test. Source data are provided as a Source Data file.

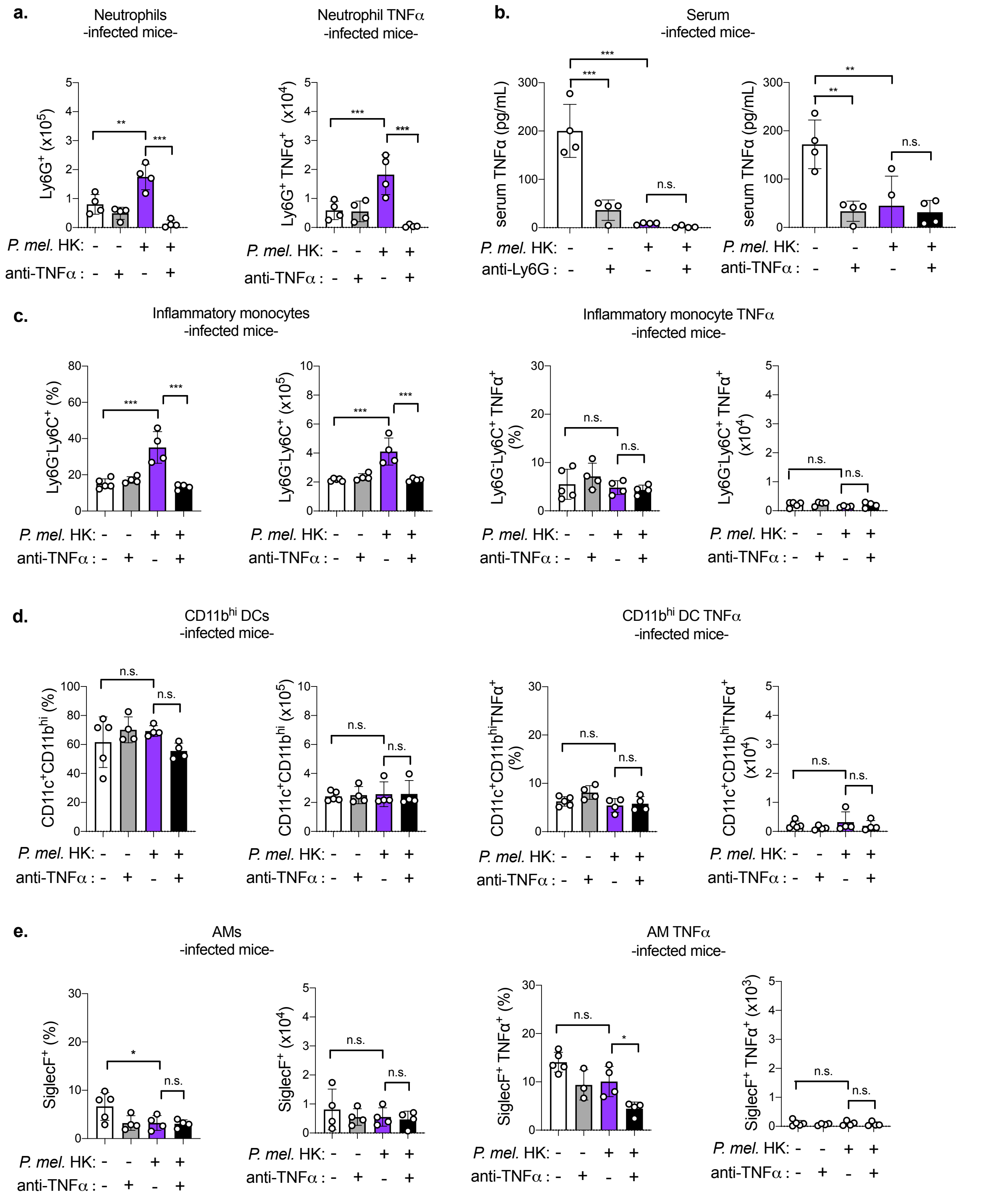


**Supplementary Figure 3.** Systemic and lung myeloid cell responses in neutrophil and TNFα depleted mice exposed to *P. melaninogenica* (*P. mel.*) prior to *S. pneumoniae* infection**. a** Total

cell number of neutrophils and neutrophil TNFα in mice treated with isotype control antibody or anti-TNFα i.p. (200 μg/mouse) together with either PBS (-) or *P. mel.* strain 25845 heat-killed (HK) i.t. prior to 24 h type 2 *S. pneumoniae* infection, $5x10^6$ CFU/mouse (n= 4 mice/group). **b** Serum TNFα detected in mice treated with isotype control antibody, anti-Ly6G or anti-TNFα i.p. (200 μg/mouse) together with either PBS (-) or *P. mel.* HK i.t. prior to 24 h *S. pneumoniae* infection (n= 4 mice/group). **c - e** Percentage and total cell number of inflammatory monocytes and inflammatory monocyte TNFα (**c**), percentage and total cell number of $CD11b^{hi}$ DCs and $CD11b^{hi}$ DC TNFα (**d**), and percentage and total cell number of alveolar macrophages (AMs) and AM TNFα (**e**), detected by intracellular flow cytometry in mice treated with isotype control antibody or anti-TNFα i.p. together with either PBS (-) or *P. mel.* HK i.t. prior to 24 h *S. pneumoniae* infection (n= 5 mice/group). Data are representative from one of four independent experiments, displayed as mean ± SEM. For (**a**) from left to right ***p*=.0018, ****p*<.0001, ****p*<.0009, ****p*<.0001, one-way ANOVA with Sidak's *post-hoc* test, (**b**) from left to right ****p*<.0001, ****p*<.0001, *p*=.9882, ***p*=.0031, ***p*=.0059, *p*=.9691, one-way ANOVA with Tukey's *post-hoc* test, (**c**) from left to right ****p*<.0001, ****p*<.0001, ****p*<.0001, ****p*=.0001, *p*=.8872, *p*=.9479, *p*=.3253, *p*=.5295, one-way ANOVA with Sidak's *post-hoc* test, (**d**) from left to right *p*=.5388, *p*=.1945, *p*=.9421, *p*=.9989, *p*=.5829, *p*=.9088, *p*=.7734, *p*=.6495, one-way ANOVA with Sidak's *post-hoc* test, (**e**) from left to right **p*=.0414, *p*=.9898, *p*=.6551, *p*=.9580, *p*=.0617, **p*=.0137, *p*=.9887, *p*=.9038, one-way ANOVA with Sidak's *post-hoc* test. Source data are provided as a Source Data file.

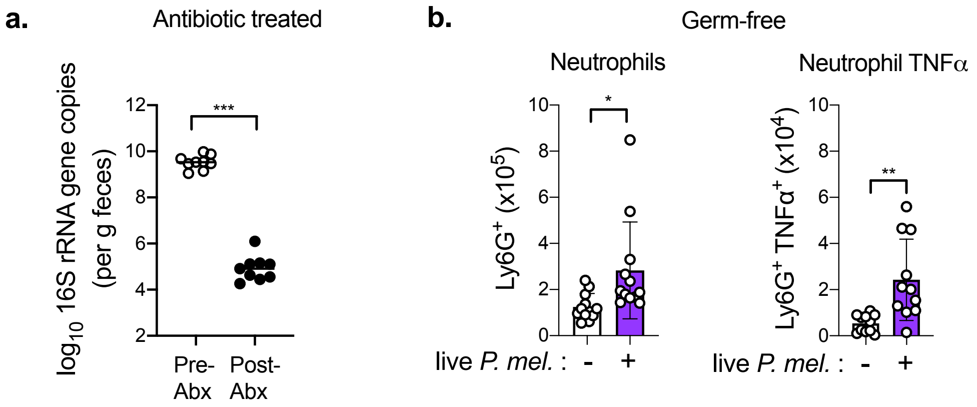


**Supplementary Figure 4**. Antibiotic depletion of the microbiome and *P. melaninogenica* (*P. mel.*)-induced neutrophil recruitment and activation in Germ-free mice. **a** 16S rRNA gene copies per gram of feces from naïve mice (pre-Abx) and following 7 days on water containing an antibiotic cocktail (post-Abx) detected by qPCR (n= 9 mice/group). **b** Total cell number of neutrophils and neutrophil TNFα detected by intracellular flow cytometry in Germ-free mice treated with either PBS (-) or live *P. mel.* strain 25845 i.t. prior to 24 h type 2 *S. pneumoniae* infection, $10^6$ CFU/mouse (n= 11 mice/group). Data are pooled three independent experiments, displayed as mean ± SEM. For (**a**) ****p*=.0004, two-tailed t test, (**b**) from left to right **p*=.0197, ***p*=.0024, two-tailed t test. Source data are provided as a Source Data file.

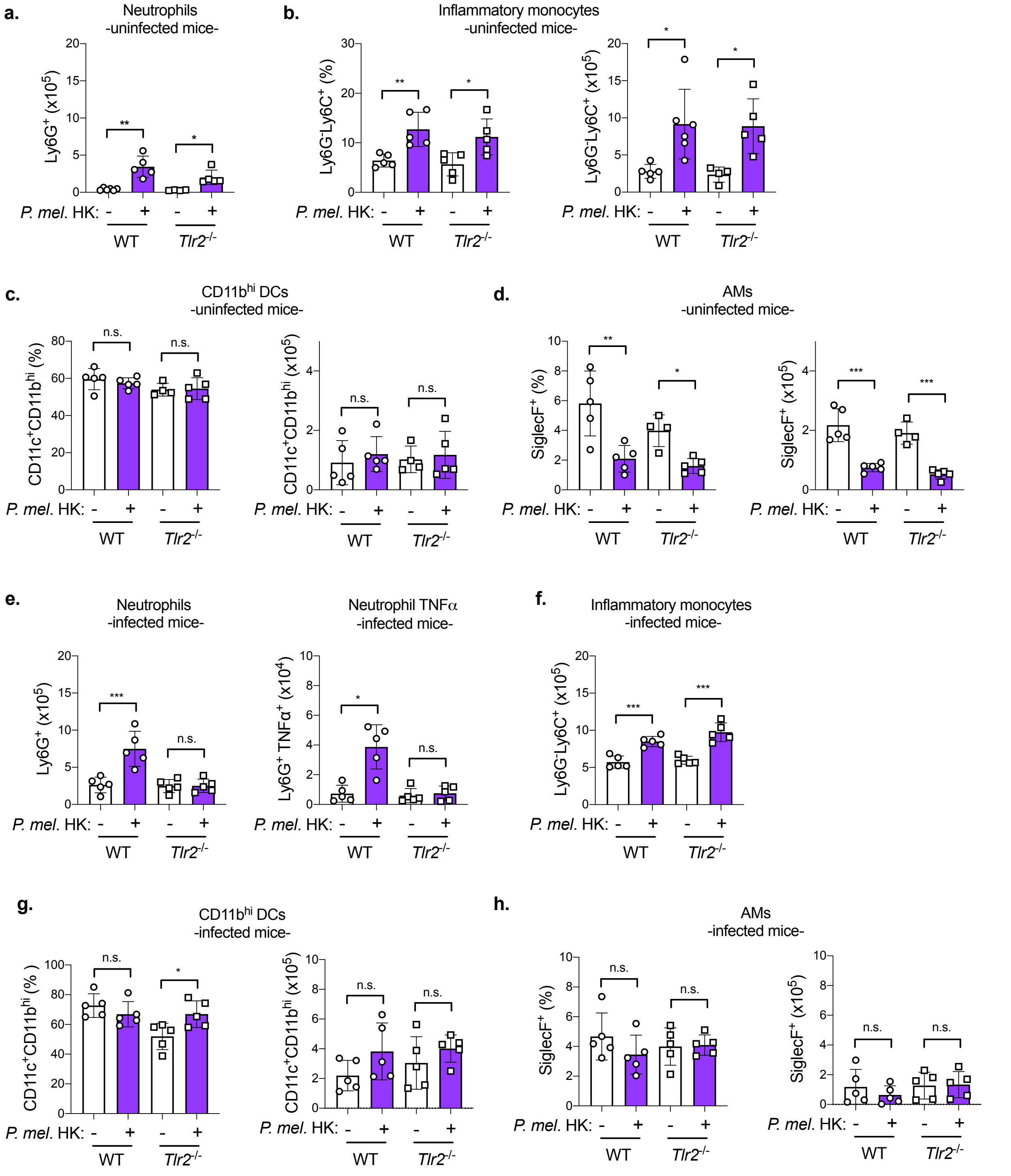


**Supplementary Figure 5.** The impact of TLR2 deficiency on the lung myeloid cell response to *P. melaninogenica* (*P. mel.*) with and without *S. pneumoniae* infection**. a** - **d** Total cell number of neutrophils (**a**), percentage and total cell number of inflammatory monocytes (**b**), percentage

and total cell number of CD11b$^{hi}$ DCs (**c**), and percentage and total cell number of alveolar macrophages (AMs) (**d**) in WT or *Tlr2*$^{-/-}$ mice treated with either PBS (-) or *P. mel.* strain 25845 heat-killed (HK) i.t. for 24 h (n= 5 mice/group for all except *Tlr2*$^{-/-}$ mice treated with PBS, n= 4 mice/group). **e - h** Total cell number of neutrophils and neutrophil TNF$\alpha$ (**e**), total cell number of inflammatory monocytes (**f**), percentage and total cell number of CD11b$^{hi}$ DCs (**g**), and percentage and total cell number of AMs (**h**) in WT or *Tlr2*$^{-/-}$ mice treated with either PBS (-) or *P. mel.* HK i.t. prior to 24 h type 2 *S. pneumoniae* infection, 5x10$^{6}$ CFU/mouse (n= 5 mice/group). Data are representative from one of four independent experiments, displayed as mean $\pm$ SEM. For (**a**) **$p$=.0040 (WT), *$p$=.0368 (*Tlr2*$^{-/-}$), Kruskal-Wallis with Dunn's *post-hoc* test, (**b**) from left to right **$p$=.0059, *$p$=.0145, *$p$=.0106, *$p$=.0177, one-way ANOVA with Sidak's *post-hoc* test, (**c**) from left to right $p$=.7207, $p$=.9819, $p$=.7592, $p$=.9317, one-way ANOVA with Sidak's *post-hoc* test, (**d**) from left to right **$p$=.0010, *$p$=.0358, ***$p$<.0001, ***$p$<.0001, one-way ANOVA with Sidak's *post-hoc* test, (**e**) from left to right ***$p$=.0001, $p$=.9990, one-way ANOVA with Sidak's *post-hoc* test, *$p$=.0279, $p$>.9999, Kruskal-Wallis with Dunn's *post-hoc* test, (**f**) ***$p$=.0002 (WT), ***$p$<.0001 (*Tlr2*$^{-/-}$), one-way ANOVA with Sidak's *post-hoc* test, (**g**) from left to right $p$=.5095, *$p$=.0273, $p$=.1896, $p$=.5296, one-way ANOVA with Sidak's *post-hoc* test, (**h**) from left to right $p$=.2665, $p$=.9912, $p$=.5916, $p$=.9922, one-way ANOVA with Sidak's *post-hoc* test. Source data are provided as a Source Data file.

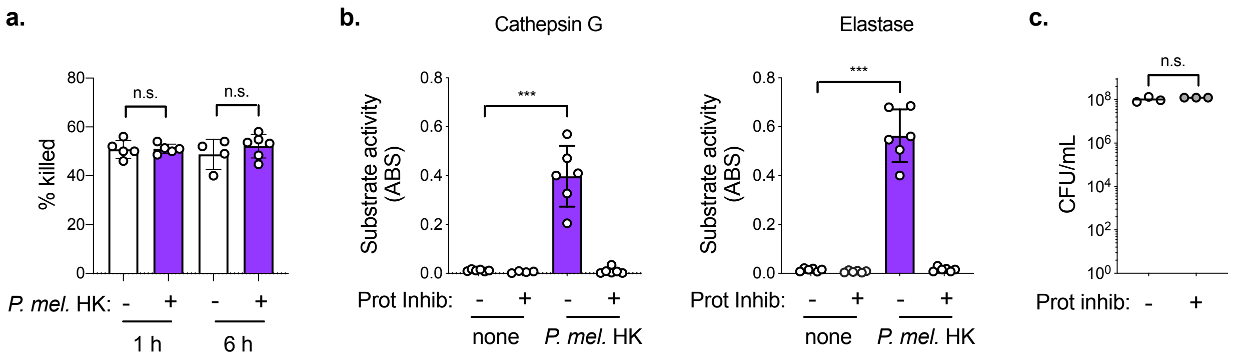


**Supplementary Figure 6.** *P. melaninogenica* (*P. mel.*) enhances serine protease-mediated killing of *S. pneumoniae* by lung neutrophils. **a** Percent of type 2 *S. pneumoniae* killed by bone marrow (BM) neutrophils from naïve mice exposed to either PBS (-) or *P. melaninogenica* (*P. mel.*) HK at a 1:1 ratio *in vitro* for 1 h or 6 h prior to incubation with *S. pneumoniae* opsonized by 3% fresh mouse serum (n= 3 independent experiments/group). **b** Serine protease activity for cathepsin G and elastase +/- protease inhibitor cocktail (Prot Inhib) detected by substrate cleavage for lung neutrophils purified from WT mice exposed to either PBS (none) or *P. mel.* heat-killed (HK) i.t. for 24 h (n= cells isolated from 6 mice/group). **c** Burden of *S. pneumoniae* detected 24 h following 1 h incubation with protease inhibitor cocktail (n= 3 independent experiments/group). Data are pooled from three independent experiments, displayed as mean ± SEM. For (**a**) $p$=.9967 (1 h), $p$=.4267 (6 h), one-way ANOVA with Sidak's *post-hoc* test, (**b**) ***$p$<.0001 (Cathepsin G), ***$p$<.0001 (Elastase), two-tailed t test, (**c**) $p$=.2573, two-tailed t test. Source data are provided as a Source Data file.

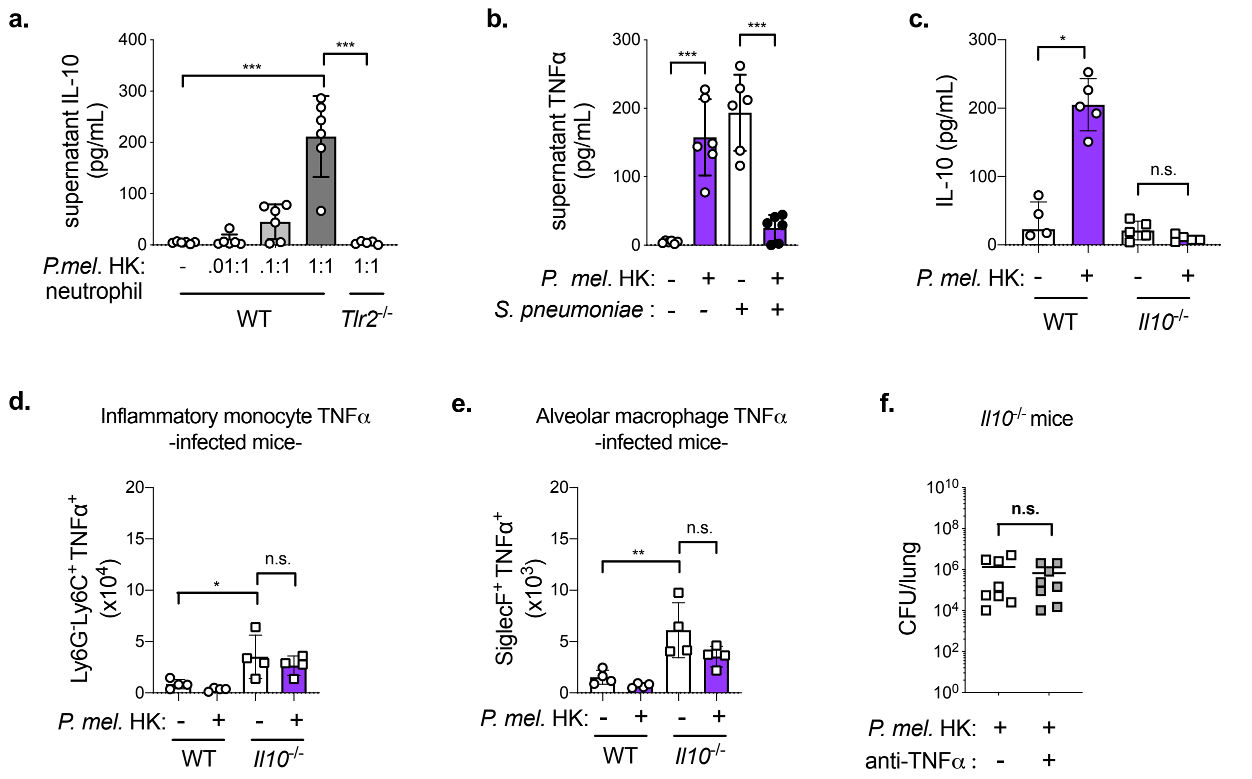


**Supplementary Figure 7.** *P. melaninogenica* (*P. mel.*) induces neutrophil IL-10 and co-infection limits neutrophil TNFα secretion**. a** Supernatant IL-10 detected 24 h following incubation of bone marrow (BM) neutrophils purified from naïve WT or *Tlr2*$^{-/-}$ mice with *P. mel.* strain 25845 heat-killed (HK) at the indicated ratios (n= 3 independent experiments/group). **b** Supernatant TNFα detected 24 h following incubation of BM neutrophils with either PBS (-), *P. mel.* HK, or infected with type 2 *S. pneumoniae* for 1 h (n= 3 independent experiments/group). **c - e** Serum IL-10 (**c**), total cell numbers of inflammatory monocyte TNFα (**d**), and total cell numbers of alveolar macrophage (AM) TNFα (**e**) detected by intracellular flow cytometry in WT or *Il10*$^{-/-}$ mice exposed to PBS (-, n= 4 mice/group) or *P. mel*. HK i.t. (n= 5 mice/group) prior to 24 h *S. pneumoniae* infection, 5x10$^{6}$ CFU/mouse. **f** Lung burdens of *S. pneumoniae* in *Il10*$^{-/-}$ mice treated with isotype control antibody or anti-TNFα (200 μg/mouse) i.p. together with *P. mel.* HK i.t. prior to 24 h *S. pneumoniae* infection (n= 8 mice/group). Data are pooled from three

independent experiments (**a - b**), representative from one of three independent experiments (**c - e**), or pooled from two independent experiments (**f**). Data are displayed as mean ± SEM. For (**a**) from left to right ***$p$<.0001, ***$p$<.0001, one-way ANOVA with Tukey's *post-hoc* test, (**b**) ***$p$<.0001, one-way ANOVA with Tukey's *post-hoc* test, (**c**) *$p$=.0431 (WT), $p$=.6196 ($Il10^{-/-}$), Kruskal-Wallis with Dunn's *post-hoc* test, (**d**) from left to right *$p$=.0151, $p$=.5387, one-way ANOVA with Sidak's *post-hoc* test, (**e**) from left to right **$p$=.0018, $p$=.0600, one-way ANOVA with Sidak's *post-hoc* test, (**f**) $p$=.8581, two-tailed Mann-Whitney U test. Source data are provided as a Source Data file.
