# Supplementary material for: Airway Prevotella promote TLR2-dependent neutrophil activation and rapid clearance of Streptococcus pneumoniae from the lung
Source: Nat Commun. 2022 Jun 9;13:3321. doi: 10.1038/s41467-022-31074-0 (PMC9184549; doi:10.1038/s41467-022-31074-0)
Supplement: Supplementary file 3 — Reporting Summary [file 41467_2022_31074_MOESM3_ESM.pdf]

nature portfolio

Corresponding author(s): Sarah E. Clark

Last updated by author(s): May 25, 2022

Reporting Summary

Nature Portfolio wishes to improve the reproducibility of the work that we publish. This form provides structure for consistency and transparency in reporting. For further information on Nature Portfolio policies, see our Editorial Policies and the Editorial Policy Checklist.

Statistics

For all statistical analyses, confirm that the following items are present in the figure legend, table legend, main text, or Methods section.

n/a	Confirmed	
☐	☒	The exact sample size (*n*) for each experimental group/condition, given as a discrete number and unit of measurement
☐	☒	A statement on whether measurements were taken from distinct samples or whether the same sample was measured repeatedly
☐	☒	The statistical test(s) used AND whether they are one- or two-sided<br>*Only common tests should be described solely by name; describe more complex techniques in the Methods section.*
☐	☒	A description of all covariates tested
☐	☒	A description of any assumptions or corrections, such as tests of normality and adjustment for multiple comparisons
☐	☒	A full description of the statistical parameters including central tendency (e.g. means) or other basic estimates (e.g. regression coefficient) AND variation (e.g. standard deviation) or associated estimates of uncertainty (e.g. confidence intervals)
☐	☒	For null hypothesis testing, the test statistic (e.g. *F*, *t*, *r*) with confidence intervals, effect sizes, degrees of freedom and *P* value noted<br>*Give P values as exact values whenever suitable.*
☒	☐	For Bayesian analysis, information on the choice of priors and Markov chain Monte Carlo settings
☒	☐	For hierarchical and complex designs, identification of the appropriate level for tests and full reporting of outcomes
☒	☐	Estimates of effect sizes (e.g. Cohen's *d*, Pearson's *r*), indicating how they were calculated

Our web collection on statistics for biologists contains articles on many of the points above.

Software and code

Policy information about availability of computer code

Data collection	No software was used
Data analysis	Flow cytometry data were analyzed using FlowJo software (version 9), LEGENDplex data were analyzed using the BioLegend LEGENDplex Data Analysis Suite (version 2022-02-10), qPCR data were analyzed using CFX Manager Software (BioRad, version 2.1), and all other data were analyzed using Prism (GraphPad, version 8) Software.

For manuscripts utilizing custom algorithms or software that are central to the research but not yet described in published literature, software must be made available to editors and reviewers. We strongly encourage code deposition in a community repository (e.g. GitHub). See the Nature Portfolio guidelines for submitting code & software for further information.

Data

Policy information about availability of data

All manuscripts must include a data availability statement. This statement should provide the following information, where applicable:

- Accession codes, unique identifiers, or web links for publicly available datasets
- A description of any restrictions on data availability
- For clinical datasets or third party data, please ensure that the statement adheres to our policy

All data supporting the findings of this study are available within the paper (and its supplementary information files). Source data are provided with this paper.

Field-specific reporting

Please select the one below that is the best fit for your research. If you are not sure, read the appropriate sections before making your selection.

☒ Life sciences ☐ Behavioural & social sciences ☐ Ecological, evolutionary & environmental sciences

For a reference copy of the document with all sections, see nature.com/documents/nr-reporting-summary-flat.pdf

Life sciences study design

All studies must disclose on these points even when the disclosure is negative.

Sample size	Sample size was chosen following a power analysis for sufficient power (80%) to detect differences between groups with a 5% significance level.
Data exclusions	Data were excluded from analysis when meeting the pre-established exclusion criteria of an infectious dose for S. pneumoniae of <10^6 CFU per mouse, as such infections are typically cleared prior to the 24 hour post-infection analysis time point used for these studies.
Replication	Experiments were replicated a minimum of three times. All attempts at data replication, aside from excluded data specified above, were successful.
Randomization	Mice were randomly allocated between groups.
Blinding	Investigators were blinded to group allocation during data analysis. Investigators were blinded to group allocation in some cases, when possible. Blinding to group allocation was not possible for some experiments where the same individual was responsible for preparing infectious materials for injection (which is visually distinguished from PBS control material) and completing all experimental procedures from start to finish.

Reporting for specific materials, systems and methods

We require information from authors about some types of materials, experimental systems and methods used in many studies. Here, indicate whether each material, system or method listed is relevant to your study. If you are not sure if a list item applies to your research, read the appropriate section before selecting a response.

Materials & experimental systems

n/a	Involved in the study	
☐	☒	Antibodies
☒	☐	Eukaryotic cell lines
☒	☐	Palaeontology and archaeology
☐	☒	Animals and other organisms
☒	☐	Human research participants
☒	☐	Clinical data
☒	☐	Dual use research of concern

Methods

n/a	Involved in the study	
☒	☐	ChIP-seq
☐	☒	Flow cytometry
☒	☐	MRI-based neuroimaging

Antibodies

Antibodies used	Flow cytometry antibodies: Siglec F (BD, catalog #562681, clone E50-2440, lot #B302914), MHCII (BioLegend, catalog #107643, clone M5/114.15.2, lot #B317262), Ly6G (BioLegend, catalog #127614, clone 1A8, lot #B292772), Ly6C (BioLegend, catalog #128012, clone HK1.4, lot #B250462), CD45.2 (BD, catalog #564616, clone 104, lot #1083734), CD11c (BioLegend, catalog #117338, clone N418, lot #B290360), CD11b (BioLegend, catalog #101212, clone M1/70, lot #B281906), and TNF-alpha (ThermoFisher Scientific, catalog #25-7321-82, clone MP6-XT22, lot #2044683). In vivo depletion antibodies from BioXCell: Ly6G (catalog #BE0075-1, clone 1A8, lot #80772101), TNF-alpha (catalog #BE0058, clone XT3.11, lot #728221A1), IgG2a isotype control (catalog #BE0085, clone C1.18.4, lot #722719J2). Fc receptor blocking antibody: CD16/32 (supernatant from 2.4G2 hybridoma, ATCC catalog #BH-197).
Validation	Antibodies targeting mouse cells for flow cytometry have been validated for this purpose by the manufacturers, with data presented on the catalog-specific references on manufacturer websites (BD, BioLegend, ThermoFisher Scientific). In vivo depletion antibodies from BioXCell have been validated by independent groups (PMID: 30266866, 30357853) and depletions were confirmed in our presented data (using flow cytometry and serum analysis).

Animals and other organisms

Policy information about studies involving animals; ARRIVE guidelines recommended for reporting animal research

Laboratory animals	Adult male and female mice (Mus musculus) aged 6-12 weeks were used for these studies. Strains included: C57BL/6J (WT), B6.129Tlr2tm1Kir (Tlr2-/-), and B6.129il10tm1Cgn (Il10-/-) mice purchased from The Jackson Laboratory (stocks #000664, 004650,

	and 002251 respectively). All strains used in these studies (WT, Tlr2-/- and Il10-/-) are on the C57BL/6J genetic background. These studies were approved by the Animal Care and Use Committee of the University of Colorado School of Medicine (protocol #00927).
Wild animals	This study did not involve wild animals.
Field-collected samples	This study did not involve samples collected from the field.
Ethics oversight	These studies were approved by the Animal Care and Use Committee of the University of Colorado School of Medicine (protocol #00927) and by the Institutional Biosafety Committee (protocol #1418).

Note that full information on the approval of the study protocol must also be provided in the manuscript.

Flow Cytometry

Plots

Confirm that:

- [x] The axis labels state the marker and fluorochrome used (e.g. CD4-FITC).
- [x] The axis scales are clearly visible. Include numbers along axes only for bottom left plot of group (a 'group' is an analysis of identical markers).
- [x] All plots are contour plots with outliers or pseudocolor plots.
- [x] A numerical value for number of cells or percentage (with statistics) is provided.

Methodology

Sample preparation	Perfused lungs were subjected to mechanical (mincing) and enzymatic (DNAseI 30 ug/mL, Sigma, and type 4 collagenase 1 mg/mL, Worthington Biochemical Corporation) digestion prior to passage through a 70 uM strainer. Red blood cells were lysed in RBC lysis buffer (0.15M NH4Cl, 10 mM KHC03, 0.1 mM Na2EDTA, pH 7.4). Fc receptors were blocked by incubation in anti-CD16/32 (2.4G2 hybridoma supernatant) prior to staining in FACS buffer (1% BSA, 0.01% NaN3, PBS). For intracellular flow cytometry, cells were incubated with Brefeldin A (BD Biosciences) prior to staining and permeabilized with 1 mg/mL saponin (Sigma) prior to intracellular staining. All cells were fixed in 1% paraformaldehyde.
Instrument	Flow cytometry was performed on an LSR Fortessa X-20 in the ImmunoMicro Flow Cytometry Shared Resource Laboratory at the University of Colorado Anschutz Medical Campus (RRID:SCR_021321).
Software	Data analysis was performed using FlowJoTM Software, version 9.9.6 (BD Life Sciences).
Cell population abundance	Flow cytometry experiments were performed on single cell isolations (unsorted), and data presented include the % and total cell numbers for each population analyzed, including AMs, neutrophils, inflammatory monocytes, and CD11bhi DCs.
Gating strategy	Cells were gated on: SSC-A x FSC-A, then FSC-W x FSC-A (single cells), then SSC-A x CD45 (CD45+ cells), then SiglecF x CD11b (for AMs), then Ly6G x CD11b (for neutrophils), then Ly6C x CD11b (for inflammatory monocytes) OR following CD45+ gate, then FSC-A x CD11c (for CD11c+ cells), then SiglecF x MHCII (for all DCs), then MHCII x CD11b (for CD11bhi DCs). Gated populations defined above were also analyzed for intracellular expression of TNFalpha using the following gates: SiglecF x TNFalpha (AM TNFalpha), Ly6G x TNFalpha (neutrophil TNFalpha), Ly6C x TNFalpha (inflammatory monocyte TNFalpha), CD11c x TNFalpha (CD11bhi DC TNF alpha).

- [x] Tick this box to confirm that a figure exemplifying the gating strategy is provided in the Supplementary Information.
